# Characterization of the clonal profile of MRSA isolated in neonatal and pediatric intensive care units of a University Hospital

**DOI:** 10.1186/s12941-014-0050-4

**Published:** 2014-11-07

**Authors:** Valéria Cataneli Pereira, Danilo Flávio Moraes Riboli, Maria de Lourdes Ribeiro de Souza da Cunha

**Affiliations:** Laboratory of Bacteriology, Department of Microbiology and Immunology, Institute of Biosciences, UNESP - Univ Estadual Paulista, Botucatu, São Paulo, CEP 18618-970 Brazil

**Keywords:** Nosocomial infections, NICU, PICU, MRSA

## Abstract

**Background:**

Methicillin-resistant *Staphylococcus aureus* (MRSA) are important pathogens in neonatal and pediatric intensive care units, which can cause severe infections in hospitalized children. Detection of the *mec*A gene and classification of the staphylococcal cassette chromosome *mec* (SCC*mec*) permit the characterization of MRSA strains isolated from infections caused by these microorganisms. In contrast, pulsed-field gel electrophoresis (PFGE) is used to type MRSA clones. This method is commonly used to analyze the epidemiology of bacteria causing nosocomial infections. The objective of this study was to detect and characterize MRSA isolated from clinical specimens of children hospitalized in the neonatal and pediatric intensive care units of the University Hospital of the Botucatu Medical School.

**Methods:**

A total of 119 *S. aureus* strains were isolated from clinical specimens and the *mec*A gene was detected by PCR. SCC*mec* was detected by multiplex PCR and the clonal profile was analyzed by PFGE.

**Results:**

The *mec*A gene was detected in 17.6% (21/119) of the isolates; 42.9% (9/21) of MRSA were characterized as SCC*mec* type III and 57.1% (12/21) as type IV. Analysis of the clonal profile of these strains revealed three distinct clones, with SCC*mec* type III being related to the Brazilian endemic clone and type IV to clones JCSC4469 and USA800.

**Conclusions:**

Replacement of clonal groups occurred in the neonatal and pediatric units over the period studied, a fact highlighting the importance of improving hygiene practices and control measures of nosocomial infections in these units.

## Background

The genus *Staphylococcus* is a member of the family Staphylococcaceae, which comprises 49 species and 26 subspecies [1,2]. *Staphylococcus aureus* is the most important species of this genus and the causative agent of a range of infections, such as furuncles, cellulitis, impetigo, and wound infections. Some of the most severe infections caused by *S. aureus* include bacteremia, pneumonia, osteomyelitis, acute endocarditis, myocarditis, meningitis, and abscesses in muscles, genitourinary tract, central nervous system and various intra-abdominal organs [3,4].

Studies have shown that 60 to 85% of staphylococci isolated from clinical samples are resistant to methicillin [5]. Methicillin-resistant *Staphylococcus aureus* strains (MRSA) are important pathogens in neonatal (NICU) and pediatric intensive care units (PICU), which can cause severe infections in hospitalized children who are generally exposed to several risk factors, such as prematurity, invasive procedures, mechanical ventilation, and drains [6].

Oxacillin is the drug of choice for susceptibility testing and treatment of infections caused by *Staphylococcus*. Intrinsic resistance of *S. aureus* to oxacillin is mediated by the production of a supplemental penicillin-binding protein (PBP 2a), which is encoded by the *mec*A gene [7]. This gene is found on a specific mobile genetic element identified as the staphylococcal cassette chromosome *mec* (SCC*mec*), which consists of the *mec*A gene complex, *ccr* gene complex, and region J. The *mec* complex comprises the *mec*A gene and its regulatory genes *mec*I and *mec*RI. The *ccr* gene complex is responsible for the integration and excision of SCC*mec* in the chromosome. In contrast, region J is not essential for the cassette chromosome, but can carry genes that encode resistance to non-beta-lactam antibiotics and heavy metals [8]. Eleven SCC*mec* types have been described so far [9]. These types are defined based on the combination of the type of *ccr* gene complex and class of the *mec* gene complex. Subtypes are defined based on polymorphisms in region J of the same combination of *mec* and *ccr* complexes [8].

SCC*mec* types I, II and III are classically found in nosocomial MRSA strains, whereas the other types are found in community-associated MRSA [10]. SCC*mec* type III encodes the largest number of resistance genes and strains harboring this type are important pathogens in hospitals where they cause severe infections [11]. In contrast, type IV is characterized by a smaller size and lower metabolic cost, a fact selectively favoring this element for transfer between staphylococci [12]. Community-associated MRSA have been reported to cause severe infections in NICU and PICU patients who never have been hospitalized [6]. According to these authors, the most frequent complications caused by these microorganisms are pneumonia and skin and soft tissue infections and strains carrying SCC*mec* type IV are the most common [6].

The increasing occurrence of MRSA in hospitals makes the typing of these microorganisms important in order to determine whether the strains involved in nosocomial infections or in possible foci of transmission are related to a specific clone, i.e., whether they have a common origin [13]. Pulsed-field gel electrophoresis (PFGE) is commonly used to analyze the epidemiology of bacteria causing nosocomial infections. This method permits to clearly discriminate strains and to demonstrate the genetic relationship between isolates with high reproducibility [13]. The objective of the present study was to detect and characterize MRSA isolated from clinical specimens of children hospitalized in the NICU and PICU of the University Hospital of the Botucatu Medical School (HC-FMB).

## Methods

### Strains

A total of 119 *S. aureus* strains isolated from clinical specimens of children hospitalized in the NICU and PICU of HC-FMB between 1991 and 2009 were studied. Thirty-nine of the 76 neonatal strains were isolated from blood cultures, 22 from secretions, 12 from catheters, and three from cannulae. In the pediatric ward, 41 of the 43 strains were isolated from blood cultures, one from pleural fluid, and one from peritoneal fluid. The strains were isolated as described by Koneman *et al.* [14] on blood agar plates (Blood Agar Base, Himedia, Mumbai, India) and suspected colonies were submitted to Gram staining. After confirmation of morphology and specific staining, the isolates were identified using catalase and coagulase tests.

### DNA extraction

Total nucleic acid was extracted from *S. aureus* isolates cultured on blood agar (Blood Agar Base, Himedia, Mumbai, India), inoculated individually into brain-heart infusion broth (Oxoid Ltd., Basingstoke, Hampshire, England), and incubated for 24 h at 37°C. Extraction was performed using the Illustra kit (Illustra™, GE Healthcare, Pittsburg, PA, USA), which consisted of initial digestion of bacterial cells with lysozyme (Amresco®, Solon, Ohio, USA) (10 mg/mL) and proteinase K (GE Healthcare, Pittsburg, PA, USA) (20 mg/mL). Five hundred μL of the extraction solution (Illustra™, GE Healthcare, Pittsburg, PA, USA) was added and the mixture was centrifuged (Centrifuge 5804 R, Eppendorf AG, Hamburg, Germany) at 5,000 × *g* for 1 min. The supernatant was transferred to a column and centrifuged (Centrifuge 5804 R, Eppendorf AG, Hamburg, Germany) at 5,000 × *g* for 1 min. The collected fluid was discarded and 500 μL extraction solution (Illustra™, GE Healthcare, Pittsburg, PA, USA) was added again to the column. After centrifugation and discarding of the collected fluid, 500 μL washing solution (Illustra™, GE Healthcare, Pittsburg, PA, USA) was added and the column was centrifuged (Centrifuge 5804 R, Eppendorf AG, Hamburg, Germany) at 20,817 × *g* for 3 min. The column was transferred to a 1.5-mL tube and 200 μL Milli-Q water heated to 70°C was used for elution. The samples were centrifuged (Centrifuge 5804 R, Eppendorf AG, Hamburg, Germany) at 5,000 × *g* for 1 min and the column was discarded. The extracted DNA was stored in a refrigerator (Brastemp BRD45, Whirlpool S.A., São Paulo, Brazil) at 4°C.

### Detection of the *mec*A gene

The *mec*A gene was investigated in the *S. aureus* isolates for detection of oxacillin resistance. The primers and parameters described by Murakami *et al*. [15] were used for amplification: primers *mec*A1 (AAA ATC GAT GGT AAA GGT TGG) and *mec*A2 (AGT TCT GCA GTA CCG GAT TTG) that amplify a fragment of 533 bp. International reference strains were included as positive (*S. aureus* ATCC 33591) and negative (*S. aureus* ATCC 25923) controls in all reactions.

### Determination of the SCC*mec* type

The SCC*mec* type was determined in the MRSA isolates by multiplex PCR. The primers and parameters described by Milheiriço *et al.* [16] were used for amplification.

### Pulsed-field gel electrophoresis

The clonal profile of the *Staphylococcus* spp. isolates was determined using the modified protocol of McDougal *et al.* [17]. The strains were inoculated into brain-heart infusion broth (Oxoid Ltd., Basingstoke, Hampshire, England) and incubated for 24 h at 37°C. The isolates were centrifuged (Centrifuge 5804 R, Eppendorf AG, Hamburg) in microtubes at 15,294 × *g* for 1 min, the supernatant was discarded, 300 μL TE solution (10 mM Tris, 1 mM EDTA, pH 8.0) was added, and the strains were kept in a water bath for 10 min at 37°C. The cells were lysed by the addition of 5 μL lysostaphin (from *Staphylococcus* lyophilized powder, Sigma-Aldrich) and vortexed (Phoenix AP-56), and 300 μL of 1.8% low-melt agarose (Agarose-Low Melt, USB Corporation, Ohio, USA) was added at 37°C. Plugs were prepared from the strains and the agarose (Agarose-Low Melt, USB Corporation, Ohio, USA) was allowed to solidify. The plugs were transferred to a 24-well plate containing 2 mL EC solution (6 mM Tris-HCl, 1 M NaCl, 100 mM EDTA, 0.5% Brij-58, 0.2% sodium deoxycholate, 0.5% sodium lauroyl sarcosinate) and incubated for 4 h at 37°C. The EC solution (6 mM Tris-HCl, 1 M NaCl, 100 mM EDTA, 0.5% Brij-58, 0.2% sodium deoxycholate, 0.5% sodium lauroyl sarcosinate) was removed and the plugs were washed four times in 2 mL TE solution (10 mM Tris, 1 mM EDTA, pH 8.0) for 30 min at 21°C.

One-third of the plug and 2 μL SmaI (Fast Digest SmaI, Thermo Scientific, Lithuania, EU) were used for the restriction of genomic DNA. For restriction, buffer without the enzyme (45 μL Milli-Q water and 5 μL of the enzyme buffer) was added to a 96-well plate and the plate was stored in a refrigerator (Brastemp BRD45, Whirlpool S.A., São Paulo, Brazil) for 30 min at 4°C. The buffer without enzyme was removed and buffer containing the enzyme (43 μL Milli-Q water, 5 μL enzyme buffer, and 2 μL of the enzyme) was added. The plate was incubated in an oven (Eletrolab 101 M/3, São Paulo, Brazil) for 6 min at 37°C. Electrophoresis was carried out in a CHEF-DR III System (BioRad Laboratories, Hercules, California USA) using 1% agarose gel (Pulsed-Field Certified Agarose, BioRad Laboratories, USA) prepared in 0.5 M TBE (0.1 M Tris, 0.08 M boric acid, 1 mM EDTA) under the following conditions: pulse times of 5 to 40 s for 21 h on a linear ramp; 6 V/cm; angle of 120°; 14°C; 0.5 M TBE as running buffer. The Lambda Ladder PFG Marker (New England BioLabs, Hitchin, United Kingdom) was used as a molecular marker. The gels were stained with GelRed (400 mL distilled, water and 30 μL GelRed) (10,000X in water, Biotium, Hayward, CA) for 1 h and photographed under UV transillumination.

The BioNumerics software, version 6.1 (Applied Maths, Belgium), was used for analysis of similarity, calculation of the Dice correlation coefficient, and construction of the dendrogram by the UPGMA method (unweighted pair group method using arithmetic averages). Band position tolerance and optimization were set at 1.25 and 0.5%, respectively. A similarity coefficient of 80% was chosen for the definition of clusters.

International clones kindly provided by Dr. Antonio Carlos Campos Pignatari, Laboratório Especial de Microbiologia Clínica, Disciplina de Infectologia, Universidade Federal de São Paulo/Escola Paulista de Medicina, and by Dr. Agnes Marie Sá Figueiredo, Universidade Federal do Rio de Janeiro, Instituto de Microbiologia Prof. Paulo de Góes, Brazil, were used as controls: USA800 (SCC*mec* IVa), JCSC 1968/CA05 (SCC*mec* IVa), JCSC 978/8/6-3P (SCC*mec* IVb), MR108 (SCC*mec* IVc), JCSC 4469 (SCC*mec* IVd), WB72/USA300 (SCC*mec* IV), USA400 (SCC*mec* IV), USA500 (SCC*mec* IV), 0SPC (SCC*mec* IV), HAR24/EMRSA 15 (SCC*mec* IV), HU25 (SCC*mec* IIIa), 85/2082 (SCC*mec* III), and ANS 46 (SCC*mec* III).

## Results

The *mec*A gene was detected in 17.6% (21/119) of the *S. aureus* isolates studied. MRSA were detected in 18.4% (14/76) of the *S. aureus* strains isolated from the NICU, including seven strains isolated from blood cultures, four from secretions, and three from catheters. Seven 16.3% (7/43) strains from the PICU carried the *mec*A gene, including six strains isolated from blood cultures and one strain isolated from pleural fluid (Table 1).

**Table 1:** **Detection of MRSA and SCC** ***mec*** **type according to hospital ward and clinical specimens**

	**Neonatal intensive care unit**				**Pediatric intensive care unit**			
	**N**	**% MRSA**	**SCC** ***mec*** **type**		**N**	**% MRSA**	**SCC** ***mec*** **type**	
			**Type III**	**Type IV**			**Type III**	**Type IV**
**Blood culture **(N = 80)	39	17.5	2	5	41	14.6	1	5
**Secretion **(N = 22)	22	18.2	3	1	0	0	0	0
**Fluid** ^**a**^ (N = 2)	0	0	0	0	2	50.0	0	1
**Foreign body** ^**b**^ (N = 15)	15	20.0	3	0	0	0	0	0

N: number of strains.

^a^Peritoneal and pleural fluid.

^b^catheter and cannula.

### Characterization of the staphylococcal cassette chromosome *mec*

The 21 *mec*A gene-positive *S. aureus* isolates were submitted to multiplex PCR for characterization of the SCC*mec* type. Nine of the 21 strains (42.9%) were classified as type III and 12 (57.1%) as type IV. Eight of the nine MRSA type III strains were isolated from clinical specimens of children hospitalized in the NICU and one in the PICU. Six of the type IV strains were isolated in the NICU and six in the PICU (Table 1).

### Evolution of oxacillin resistance in *S. aureus* strains isolated from patients seen at HC-FMB

Analysis of the period from 1991 to 2009 showed the early presence of SCC*mec* type IV in a strain isolated in 1993. Although the sample size of this study was too small to detect a significant difference, the results showed a decrease in the prevalence of SCC*mec* type III and a recent increase in SCC*mec* type IV-carrying isolates.

### Analysis of the clonal profile of MRSA

**Figure 1:**
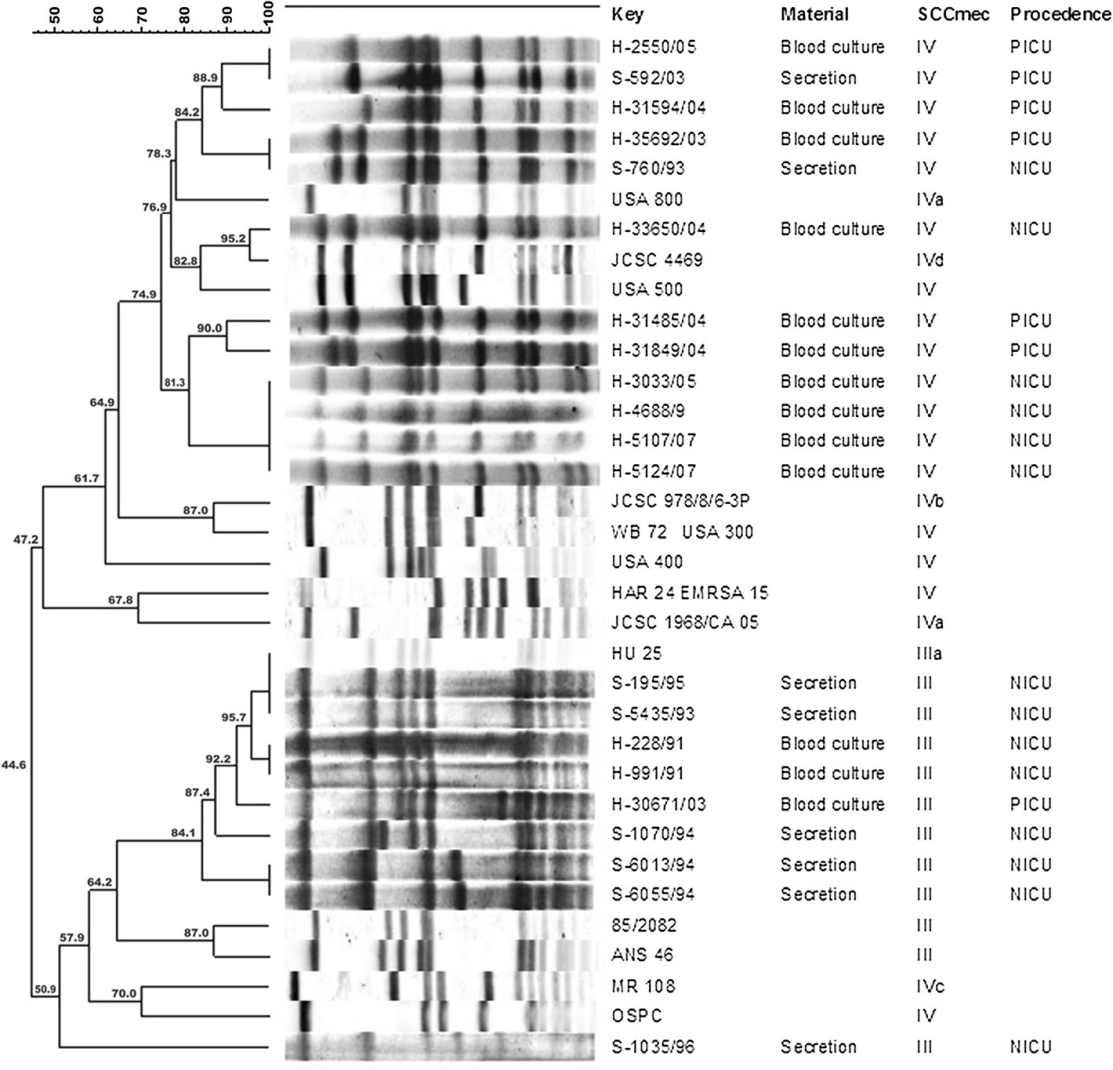
**Determination of the clonal profile of MRSA carrying SCC** ***mec*** **type III and type IV isolated from clinical specimens of children hospitalized in the neonatal (NICU) and pediatric (PICU) intensive care units of HC-FMB.**

Analysis of the clonal profile of the MRSA strains isolated in this study revealed four distinct clones. MRSA harboring SCC*mec* type III were divided into two groups, one related to the Brazilian endemic clone (HU25). The strains carrying SCC*mec* type IV were also divided into two groups, one related to a clone found in the United States (USA800) and the other related to a clone found in Japan (JCSC4469) (Figure 1).

## Discussion

Oxacillin-resistant *Staphylococcus aureus* are important pathogens involved in infections that affect children hospitalized in intensive care units in many countries. Although the frequency of oxacillin resistance is high among *S. aureus* strains, particularly in large hospitals and universities, the frequency of isolation of MRSA in the NICU and PICU of HC-FMB was 17.6% (21/119) over a period of 18 years; 18.4% (14/76) of these isolates were detected in the NICU and 16.3% (7/43) in the PICU. Similar results have been reported in a study conducted in the United Kingdom, in which *S. aureus* strains isolated in the NICU and PICU of a hospital over a period of 10 years (1993 to 2003) were analyzed. The frequency of isolation of MRSA related to bacteremia was 15.1% (5/33) [18]. In a study conducted in New Zealand, the frequency of isolation of MRSA was 12.0% (7/58) in a PICU over a period of 11 years (1993 to 2004) [19]. In contrast, different results were found in the NICU of a hospital in the United States where 47.4% (8/17) of MRSA were detected among *S. aureus* over a period of 10 years (2000 to 2009) [20].

In the two wards, the *S. aureus* strains isolated from blood cultures exhibited a similar percentage of oxacillin resistance [NICU: 17.5% (7/39), PICU: 14.6% (6/41)]. In the NICU, MRSA were also isolated from other clinical specimens such as secretions [18.2% (4/22)] and catheters and cannula [20.0% (3/15)]. In the PICU, only one strain isolated from pleural fluid was resistant to oxacillin.

Among the MRSA detected in this study, 57.1% (12/21) were characterized as SCC*mec* type IV; of these, 83.3% (10/12) were isolated from blood cultures. In the study of Healy *et al.* [21], 75% (6/8) of MRSA strains isolated in the NICU were typed as SCC*mec* type IV. SCC*mec* type IV is the most frequent type found in the community and is also becoming predominant among healthcare-associated MRSA infections [8,22,23]. The smaller size of the cassette chromosome when compared to types I, II and III probably increases its mobility and transfer capacity between *Staphylococcus*, suggesting that clones carrying this SCC*mec* element may spread more easily and that diseases caused by these strains tend to increase [24,25]. According to Dolapo *et al.* [20], the incidence of MRSA infections in NICUs is still unacceptably high. This fact may be related to the acquisition of community-associated MRSA strains, which have evolved in the community and penetrated the NICU through parents or care providers.

SCC*mec* type III was identified in 42.9% (9/21) of MRSA and predominated among strains isolated in the 1990s. Only one strain was detected after 2000. SCC*mec* type III is commonly found in Brazilian hospitals and is highly resistant to various antimicrobial agents used to treated *S. aureus* infections, including resistance to beta-lactams, macrolides, aminoglycosides and trimethoprim–sulfamethoxazole [26]. In the study of Perez & D’Azevedo [27], nine MRSA were susceptible only to vancomycin, linezolid and teicoplanin. Eight of these strains carried SCC*mec* type III.

In the present study, SCC*mec* typing permitted to confirm the isolation of two types of MRSA in the NICU and PICU of HC-FMB over a period of 18 years. One important finding was the isolation of MRSA carrying SCC*mec* type IV in 1993 from the secretion sample of a newborn. SCC*mec* type IV was only typed again 10 years later in the pleural fluid sample of a child hospitalized in the pediatric unit. From that time on, this SCC*mec* was the predominant type among all MRSA isolated in the two units. According to Milheiriço *et al.* [16], the SCC*mec* element is an important marker for the determination of MRSA clones. In addition to being a valuable tool for the study of MRSA epidemiology, SCC*mec* characterization permits to investigate the evolution of MRSA clones in culture collections.

With respect to the epidemiology and evolution of MRSA clones, PFGE permitted a better analysis of the data obtained in this study. The MRSA isolates carrying SCC*mec* type III were divided into two groups, one of them related to the Brazilian endemic clone (HU25). According to Vivone *et al.* [28], this clone is responsible for most infections caused by MRSA. The MRSA isolates carrying SCC*mec* type IV could also be divided into two groups, one related to clone JCSC4469 and the other related to clone USA800. The strain mentioned above, which was isolated in 1993 and carried SCC*mec* type IV, was related to clone USA800. This group comprised strains isolated between 1993 and 2005. Trindade *et al.* [29] found a variety of MRSA that were related to the Brazilian endemic clone. In the present study, strains related to the Brazilian endemic clone predominated until 2003, whereas strains related to clones JCSC4469 and USA800 were found after this period. A Brazilian study conducted in a university hospital that analyzed clonal groups over a period of 8 years found that the clones identified were replaced over time, without any predominance in a specific hospital area [30]. According to the authors, replacement of clonal groups over time might be explained by microevolution of the pathogen or by competition to adapt to the hospital environment. Furthermore, the report of the presence of the pediatric clone in central Brazil suggests that this clone is settling in Brazilian hospitals and spreading in the community, increasing the likelihood of expanding its reservoir [31].

## Conclusions

The clonal MRSA groups found in the NICU and PICU of HC-FMB highlight the importance of improving hygiene practices and control measures of nosocomial infections in these units since hospitalized children are generally more vulnerable because of exposure to several risk factors. Furthermore, the clonal groups that predominated over the past years carry SCC*mec* type IV, an element that does not impose any metabolic cost on the host and that may spread in the absence of antibiotic selective pressure. This fact may result in the emergence of this type as a new pathogen in the world. Although the sample size of this study was too small to draw any definite conclusions, according to the literature, community-associated MRSA are steadily increasing and may replace or be the more dominant population in clinical settings.

## Abbreviations

MRSA: Methicillin-resistant *Staphylococcus aureus*
NICU: Neonatal intensive care units
PICU: Pediatric intensive care units
PBP 2a: Penicillin-binding protein
SCC*mec*: Staphylococcal cassette chromosome *mec*
PFGE: Pulsed-field gel electrophoresis
HC-FMB: University hospital of the Botucatu medical school
